# Identification and functional analysis of the SARS-COV-2 nucleocapsid protein

**DOI:** 10.1186/s12866-021-02107-3

**Published:** 2021-02-22

**Authors:** Tianyi Gao, Yingdong Gao, Xiangxiang Liu, Zhenlin Nie, Huilin Sun, Kang Lin, Hongxin Peng, Shukui Wang

**Affiliations:** 1 Department of clinical Laboratory, Nanjing First Hospital, Nanjing Medical University, 68 Changle Road, Nanjing, 210006 Jiangsu China; 2 Central Laboratory, Nanjing First Hospital, Nanjing Medical University, 68 Changle Road, Nanjing, 210006 Jiangsu China; 3 Jiangsu Collaborative Innovation Center on Cancer Personalized Medicine, Nanjing Medical University, 68 Changle Road, Nanjing, 210006 Jiangsu China

**Keywords:** SARS-COV-2, Nucleocapsid protein (N protein), Structure, Phosphorylation

## Abstract

**Background:**

A severe form of pneumonia, named coronavirus disease 2019 (COVID-19) by the World Health Organization is widespread on the whole world. The severe acute respiratory syndrome coronavirus 2 (SARS-CoV-2) was proved to be the main agent of COVID-19. In the present study, we conducted an in depth analysis of the SARS-COV-2 nucleocapsid to identify potential targets that may allow identification of therapeutic targets.

**Methods:**

The SARS-COV-2 N protein subcellular localization and physicochemical property was analyzed by PSORT II Prediction and ProtParam tool. Then SOPMA tool and swiss-model was applied to analyze the structure of N protein. Next, the biological function was explored by mass spectrometry analysis and flow cytometry. At last, its potential phosphorylation sites were analyzed by NetPhos3.1 Server and PROVEAN PROTEIN.

**Results:**

SARS-COV-2 N protein composed of 419 aa, is a 45.6 kDa positively charged unstable hydrophobic protein. It has 91 and 49% similarity to SARS-CoV and MERS-CoV and is predicted to be predominantly a nuclear protein. It mainly contains random coil (55.13%) of which the tertiary structure was further determined with high reliability (95.76%). Cells transfected with SARS-COV-2 N protein usually show a G1/S phase block company with an increased expression of TUBA1C, TUBB6. At last, our analysis of SARS-COV-2 N protein predicted a total number of 12 phosphorylated sites and 9 potential protein kinases which would significantly affect SARS-COV-2 N protein function.

**Conclusion:**

In this study, we report the physicochemical properties, subcellular localization, and biological function of SARS-COV-2 N protein. The 12 phosphorylated sites and 9 potential protein kinase sites in SARS-COV-2 N protein may serve as promising targets for drug discovery and development for of a recombinant virus vaccine.

**Supplementary Information:**

The online version contains supplementary material available at 10.1186/s12866-021-02107-3.

## Background

On February 12, 2020, the World Health Organization officially named the new coronavirus causing the pneumonia epidemic in Wuhan as Coronavirus Disease 2019 (COVID-19) [1]. As of September 17, 2020, there were approximately 30,055,710 confirmed cases and 943,433 deaths in the worldwide [2]. The latest research shows that the impact of COVID-19 has far exceeded the impact of severe acute respiratory syndrome (SARS) in 2003 [3, 4]. At present, there are no clinically validated SARS-COV-2 vaccine candidates or therapeutic antibodies to prevent infection, and its diagnosis is still based on viral nucleic acid detection and false negative cases pose a problem [5]. In response to the COVID-19 outbreak, searching for potential viral genetic or protein information as soon as possible will greatly help clinicians improve diagnosis and treatment efficiency and aid in subsequent vaccine development.

The Coronaviridae family is made up of two subfamilies: Letovirinae and Orthocoronavirinae. The Orthocoronavirinae family consists of the α-coronavirus, β-coronavirus, γ-coronavirus, and δ-coronavirus genera [6]. Among them, β-coronaviruses are human which usually cause severe respiratory diseases, including SARS-CoV, the Middle Eastern Respiratory Syndrome Coronavirus (MERS-CoV), and currently, SARS-CoV-2. Coronaviruses are enveloped, positive-sense, singlestranded RNA viruses with mammalian and avian hosts. The length of the SARS-CoV-2 genome is approximately 30 kb and it encodes at least 29 proteins, including 16 non-structural proteins (NSP), 9 accessory proteins and 4 structural proteins such as (spike [S] glycoprotein, envelope [E] protein, membrane [M] protein, and nucleocapsid [N] protein [7].

The coronavirus N protein is an important viral structural protein, which plays an important role in promoting of genome packaging, RNA chaperoning, intracellular protein transport, DNA degradation, interference in host translation, and restricting host immune responses [8]. It is reported that coronavirus N protein may help tether the genome to replicase-transcriptase complex (RTC), and package the encapsidated genome into virions by binding nsp3 protein which is also an antagonist of interferon and viral encoded repressor (VSR) of RNA interference (RNAi) that further benefits the viral replication [9]. The SARS-CoV N protein, the most abundant protein in the virus infected cells, is also proved to be a genetically stable protein, which is a primary requirement for an efficient drug target candidate [10]. Phylogenetic analysis of the Severe Acute Respiratory Syndrome Coronavirus 2 (SARS-CoV-2) determined that it is most closely related (89.1% nucleotide similarity similarity) to SARS-CoV that had a history of genomic recombination [11]. The N protein of SARS-COV-2 may also be an important part of virus on host specificity and evolution of the interactions between N and host cell proteins.

To date, little is known about SARS-COV-2 N protein. Our aim is to conduct a bioinformatic analysis of the primary, secondary and tertiary structure of SARS-COV-2 N protein to inform the research community about potential targets for development of anti-viral agents.

## Results

### The sequence and location of SARS-COV-2 N protein

The complete N protein sequence was analyzed using NCBI protein-blast which showed that the SARS-COV-2 N was composed of 419 amino acids which had a 91 and 49% similarity to SARS-CoV and MERS-CoV N proteins. Subcellular localization analysis predicted that the protein had a predominantly nuclear distribution although it is present to some extent in the cytoplasm and cell membrane (k = 23, Table 1). At the same time, we also found that a small amount of protein is also predicted to be distributed to the cell vesicles, suggesting that SARS-COV-2 may be spread in the human body through the cell vesicles.

**Table 1:** The SARS-COV-2 N protein physicochemical analysis and subcellular localization

Project Name	Results
**Formula**	C_1971_H_3137_N_607_O_629_S_7_
**Number of amino acids**	419
**Molecular weight**	45.6 kDa
**Theoretical pI**	10.07
**Number of charged residues**	
Positively (Arg + Lys + His)	60
Negatively (Asp + Glu)	36
**Estimated half-life**	
Mammalian reticulocytes	30 h (vitro)
Yeast	> 20 h (vivo)
Escherichia coli	> 10 h (vivo)
**Instability index**	55.09
**Aliphatic index**	52.53
**GRAVY**	− 0.971
**Subcellular localization (KNN)**	
Nuclear	69.6%
Cytoplasmic	21.7%
Cytoskeletal	4.3%
Vesicles of secretory system	4.3%

### The physicochemical properties of SARS-COV-2 N protein

As showed in Table 1, we further studied the SARS-COV-2 N protein physicochemical properties through ProtParam tool which demonstrated it was a 45.6 kDa positively charged (PI> 10) and unstable (instability index < 60) protein. Its aliphatic index and GRAVY which was less than 70 and 0 indicated that it was also a hydrophobic protein with poor heat resistance.

### The structure of SARS-COV-2 N protein

**Fig. 1:**
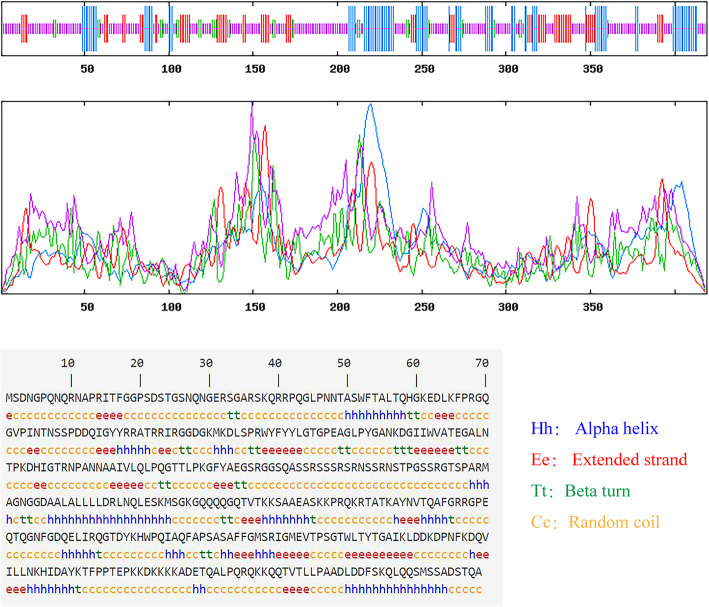
The predicted secondary structure of SARS-COV-2 N protein. SARS-COV-2 N protein is composed of alpha helix (21.24%), beta fold (16.71%), beta turn (6.92%), and random coil (55.13%). Their corresponding positions on the protein sequence are coloured blue, red, green, and yellow

The secondary structure of SARS-COV-2 N protein was predicted using window width 17, similarity threshold 8 and 4 of states which is showed in Fig. 1. The results indicated that the SARS-COV-2 N protein was made up of alpha helix (21.24%), beta fold (16.71%), beta turn (6.92%), and random coil (55.13%). As showed on Tables 2, 231 of 419 amino acid residues localized to the random coil indicating that it might be the main secondary structure of SARS-COV-2 N protein. In addition, the secondary structure of SARS-COV-2 N protein was further compared with SARS-CoV and MERS-CoV proteins which all of them showed a high similarity to each other (supplymentary Table 1).

**Table 2:** The results of SARS-COV-2 N protein tertiary structure analysis

Project Name	Results
**secondary structure**	
Alpha helix	89/419 (21.24%)
Extended strand	70/419 (16.71%)
Beta turn	29/419 (6.92%)
Random coil	231/419 (55.13%)
**Tertiary structure**	
**swiss-model**	
Seq Identity	95.76%
GMQE	0.17
QMEAN	−2.02
**SAVES v5.0**	
**ERRAT**	
Overall quality factor	93.5
**Prove**	
Z − score mean	0.833
Z − score stddev	1.336
Z − score RMS	1.572
scored atoms	230
outliers	16
% outliers	7.000
**Whatcheck**	
RMS Z-scores	
Bond lengths	0.702
Bond angles	1.057
Omega angle restraints	0.899
Side chain planarity	1.407
Structure Z-scores	
1st generation packing quality	−4.798 (poor)
Ramachandran plot appearance	−3.262 (poor)
chi-1/chi-2 rotamer normality	−3.110 (poor)
Backbone conformation	−28.233 (bad)
**Verify 3D**	59.32%
**Procheck**	87%

**Fig. 2:**
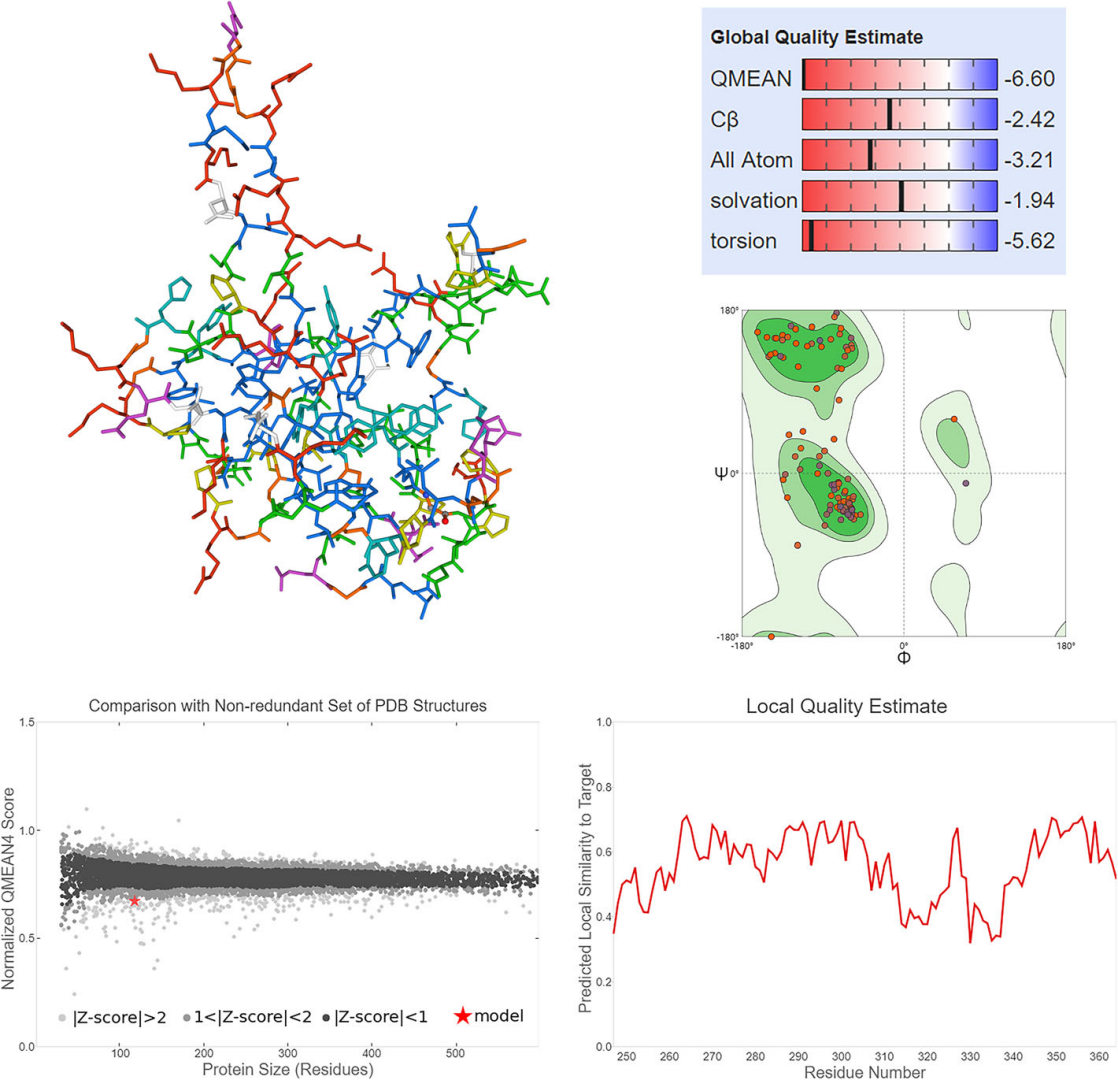
The tertiary structure model of SARS-COV-2 N protein. Colours on SARS-COV-2 N protein 3D model represent for the 419 amino acids. The global quality estimate results showed that most scores were higher than −4.0 which represents a high quality of the tertiary structure; Then Ramachandran analysis was performed which showed a 91.38% score (> 90%); Moreover, the QMEAN and local quality estimate scores were also calculated which were 0.17 and higher than 50% similarity to target

**Fig. 3:**
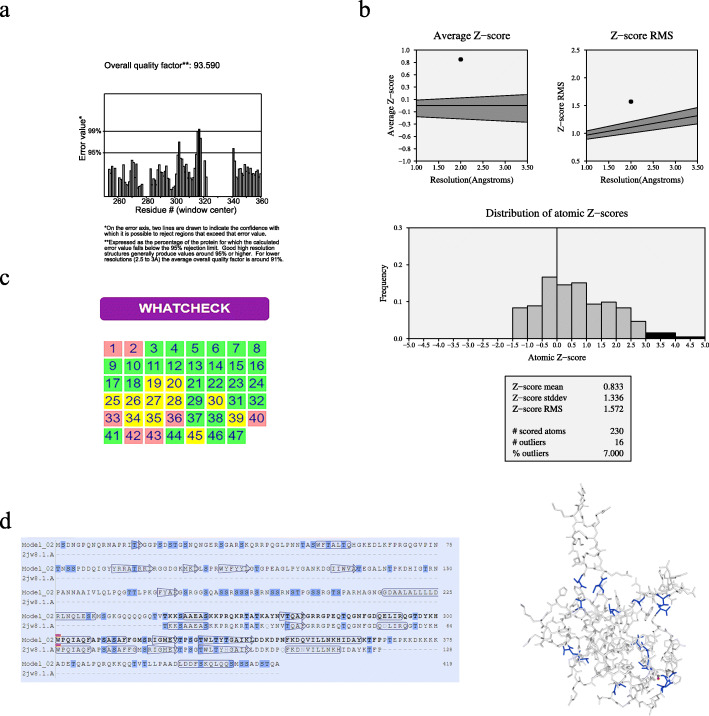
Systemic evaluation of potential phosphorylated sites in the SARS-COV-2 N protein tertiary structure. **a**: the ERRAT analysis of SARS-COV-2 N protein tertiary structure. Overall quality factor was 93.5 higher than 90; **b**: the Prove z-score of SARS-COV-2 N protein tertiary structure; **c**: the analysis results of Whatcheck on SARS-COV-2 N protein tertiary structure. The favorable results are colored green which were significantly higher than 50%; **d**: the distribution of phosphorylated sites on SARS-COV-2 N protein. The phosphorylated sites were colored blue on the SARS-COV-2 N protein tertiary structure

With the help of Swiss-model, the tertiary structure was constructed with a 95.76% sequence identity (Fig. 2). The SAVES v5.0 that contains more than 5 different verification methods was further performed to verify the tertiary structure model of SARS-COV-2 N protein (Table 2). AThe overall quality factor of ERRAT was higher than 90(Fig. 3a), the z-score of Prove was close to 1(Fig. 3b). Whatcheck analysis of expected properties showed green positive results which confirmed the usefulness of our SARS-COV-2 N protein tertiary structure model (Fig. 3c)..

### The biological function of SARS-COV-2 N protein on cell cycle

**Fig. 4:**
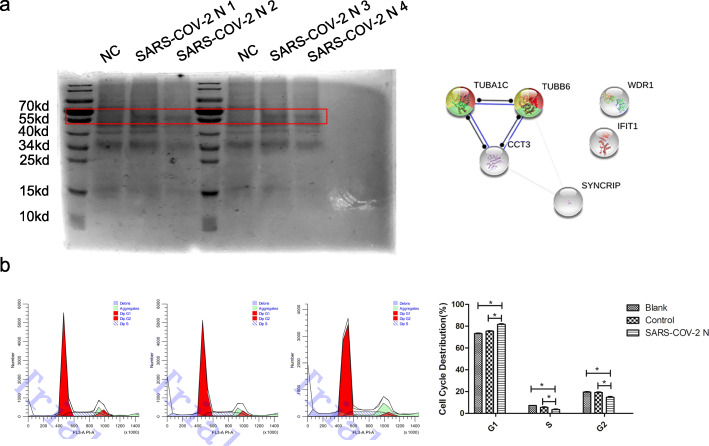
The exploration of SARS-COV-2 N proteins’ biological function on host cells. **a**: western-blot results of host cells transfected with SARS-COV-2 N plasmid and negative control before mass spectrometry analysis. A significant higher expression of TUBA1C, IFIT1, TUBB6, CCT3, WDR1, SYNCRIP protein was found between 40kd and 70kd; **b**: The cell cycle results of host cells transfected with SARS-COV-2 N plasmid or negative control and cells without any treatment using flow cytometry. *: *p* < 0.05

As showed on Fig. 4a, Cells transfected with SARS-COV-2 N protein or negative control were detected with mass spectrometry analysis. A significant higher expression of TUBA1C, IFIT1, TUBB6, CCT3, WDR1, SYNCRIP protein was found on SARS-COV-2 N protein transfection group comparing with negative control. Then the six proteins were predicted by STRING database. We fortunately found that high expression of TUBA1C, TUBB6 might be related to the Cell Cycle, Mitotic regulation (*p* = 0.0199). Therefore, the cell cycle analysis was performed. The results showed that host cells transfected with SARS-COV-2 N plasmid had higher rates on G1 phase and lower rates on S or G2 phase than other groups which demonstrated a G1/S cycle was blocked for the affection of SARS-COV-2 N protein (Fig. 4b, *p* < 0.05).

### The SARS-COV-2 N protein phosphorylated sites prediction

A total number of 56 phosphorylated sites were identified in SARS-COV-2 N protein (Fig. 3d). Only 46 of them showed probable protein kinase except unsp. Finally, 18 phosphorylated sites with specific predicted kinases were found in the remaining 46 sites (Table 3) and 9 protein kinases such as PKA, PKC, PKG, EGFR, DNAPK, CKI, CKII, CDC2, ATM were predicted to be the main kinase involved in SARS-COV-2 N protein phosphorylation. Next, to further confirm the phosphorylated sits impact on the biological function of N protein, PROVEAN PROTEIN were performed which demonstrated that single amino acid substitution of 87Y, 115 T would significantly changed the biological function of N protein. 57 T, 87Y, 115 T, 255S, 263 T, 265 T, 271 T, 332 T deletion would lead to a significant broken of N protein function. Single amino acid insertion affect N protein function was also found on 87Y, 115 T, 263 T, 265 T, 271 T. At last, the replacements of phosphorylated sites were explored which declared 87Y, 115 T, S176, 265 T, 271 T, 332 T should play an important role on SARS-COV-2 N protein function (Table 3). Unfortunately, positive results were not found on the phosphorylated sites 49 T, 232S, 245 T, 366 T, 379 T, 391 T, 393 T, 413S, 417 T. Therefore, a further analysis of multiple amino acids variant affection was applied which 49 T, 245 T, 366 T showed a significant impaction on protein function (Table 4). 232S, 379 T, 391 T, 393 T, 413S, 417 T might not be the main amino acids on SARS-COV-2 N protein function.

**Table 3:** The prediction of SARS-COV-2 N protein phosphorylation site single amino acid variant

Sites	Kinases	Substitution			Deletion			Insertion			Replacements		
		Variant	score	Prediction	Variant	score	Prediction	Variant	score	Prediction	Variant	score	Prediction
49 T	CDC2	T49A	−0.798	N^a^	T49del	−1.499	N	T49_A50insVA	−1.795	N	T49_A50delinsVA	−0.938	N
57 T	PKC,CKI	T57A	− 1.479	N	T57del	−3.366	D	T57_Q58insVA	−0.768	N	T57_Q58delinsVA	−1.973	N
87Y	EGFR	Y87A	−4.899	D	Y87del	−3.899	D	Y87_R88insVA	−4.820	D	Y87_R88delinsVA	−5.600	D
115 T	PKC,CKII	T115A	−4.678	D	T115del	−14.005	D	T115_G116insVA	−10.362	D	T115_G116delinsVA	−10.295	D
176S	CDC2	S176A	−1.956	N	S176del	−1.373	N	S176_R177insVA	−0.436	N	S176_R177delinsVA	−4.134	D
232S	PKC,CDC2	S232A	−0.372	N	S232del	−1.402	N	S232_K233insVA	−2.151	N	S232_K233delinsVA	−0.595	N
245 T	PKC	T245A	−0.005	N	T245del	−1.055	N	T245_V246insVA	−2.157	N	T245_V246delinsVA	−2.021	N
255S	PKC	S255A	0.397	N	S255del	−2.807	D	S255_K256insVA	−2.195	N	S255_K256delinsVA	−2.067	N
263 T	PKG	T263A	−2.227	N	T263del	−8.631	D	T263_A264insVA	−6.701	D	T263_A264delinsVA	−0.967	N
265 T	PKA	T265A	−0.866	N	T265del	−6.947	D	T265_K266insVA	−6.914	D	T265_K266delinsVA	−4.805	D
271 T	DNAPK	T271A	−2.049	N	T271del	−9.555	D	T271_Q272insVA	−7.831	D	T271_Q272delinsVA	−4.574	D
332 T	CKI	T332A	−1.844	N	T332del	−6.020	D	T332_Y333insVA	−1.285	N	T332_Y333delinsVA	−8.550	D
366 T	PKC	T366A	−0.363	N	T366del	−0.649	N	T366_E367insVA	−2.147	N	T366_E367delinsVA	−2.494	N
379 T	DNAPK	T379A	0.352	N	T379del	−0.572	N	T379_Q380insVA	−0.004	N	T379_Q380delinsVA	−0.160	N
391 T	PKA	T391A	0.231	N	T391del	−0.123	N	T391_V392insVA	−0.163	N	T391_V392delinsVA	0.414	N
393 T	CDC2	T393A	−0.755	N	T393del	−0.356	N	T393_L394insVA	−0.188	N	T393_L394delinsVA	0.632	N
413S	CDC2	S413A	0.470	N	S413del	1.119	N	S413_A414insVA	0.998	N	S413_A414delinsVA	0.625	N
417 T	DNAPK,ATM	T417A	−0.210	N	T417del	−0.226	N	T417_Q418insVA	0.433	N	T417_Q418delinsVA	−0.212	N

^**a**^**Default threshold is − 2.5, that is: Variants with a score equal to or below − 2.5 are considered “Deleterious”; Variants with a score above − 2.5 are considered “Neutral”**

**Table 4:** The analysis of SARS-COV-2 N protein phosphorylation site multiple amino acid variant

Sites	Deletion			Insertion			Replacements		
	Variant	score	Prediction	Variant	score	Prediction	Variant	score	Prediction
49 T	T49_S51del	−4.946	D^a^	T49_S51dup	−1.515	N	T49delinsSW	−3.009	D
232S	S232_M234del	−1.561	N	S232_M234dup	−1.573	N	S232delinsMS	−1.350	N
245 T	T245_T247del	−5.955	D	T245_T247dup	−3.081	D	T245delinsTK	−0.686	N
366 T	T366_P368del	−3.160	D	T366_P368dup	−0.983	N	T366delinsPK	−0.985	N
379 T	T379_A381del	−1.021	N	T379_A381dup	0.060	N	T379delinsAL	−1.584	N
391 T	T391_T393del	−0.007	N	T391_T393dup	−0.175	N	T391delinsTL	−1.299	N
393 T	T393_L395del	0.779	N	T393_L395dup	−1.736	N	T393delinsLP	−1.429	N
413S	S413_D415del	−1.187	N	S413_D415dup	1.058	N	S413delinsDS	1.891	N
417 T	T417_A419del	0.182	N	T417_A419dup	0.488	N	T417delinsAA	0.420	N

^**a**^**Default threshold is − 2.5, that is: Variants with a score equal to or below − 2.5 are considered “Deleterious”; Variants with a score above − 2.5 are considered “Neutral”**

## Discussion

In order to thoroughly control the spread of SARS-COV-2 and design reasonable drugs for prevention and treatment, we must first understand the biological functions of SARS-COV-2 structure protein. In this study, we primary analyzed the SARS-COV-2 N protein which was a 45.6 kDa, positively charged, unstable hydrophobic protein with poor heat resistance protein mainly composed of 419 amino acid residues.

The coronavirus N protein could cause deregulation of the cell-cycle which offered a better environment for itself binding to viral RNA to form the ribonucleocapsid and promoting virus replication, transcription and translation [12]. Study showed the reason of it might due to its localization to the nucleolus [13]. The later study of the SARS-CoV and MERS N protein function also confirm the interaction with nucleic acids. In this study, we found that the SARS-COV-2 N protein might located mainly on nuclear and had a 91 and 49% similarity to SARS-CoV and MERS-CoV not only protein sequence but also secondary structure which indicated that SARS-COV-2 N protein should also play an important role on SARS-COV-2 replication.

The phosphorylation of virus proteins can regulate their activity, localization and interactions with host intracellular proteins which is an important sign of active viral replication [14]. Moreover, the phosphorylation of coronavirus N protein was reported to played an important role on its localization and interactions with host cell nucleolus which could further delay the cell cycle and creates a mechanism that is conducive to viral RNA translation [15, 16]. Studies on SARS-CoV showed its N protein phosphorylation was significantly correlated to nucleoplasmic shuttle capacity which might further block host cell G1/S phase [10, 13]. In this study, a significant G1/S phase was also observed on cells tranfected with SARS-COV-2 N protein. Furthermore, a total number of 12 phosphorylation sites were identified on SARS-COV-2 N protein and analyzed to be significantly associated with N protein functions.

The researches exposed that microtubules combined with many microtubule-related proteins such as g α-, β-, and γ-tubulin aggregate to achieve various cellular functions in the cell cycle (mitosis and meiosis) [17, 18]. Moreover, TUBA1C, a subtype of α-tubulin, which is composed of microtubule structure, was reported to be overexpressed and promotes oncogenesis in pancreatic ductal adenocarcinoma via Regulating the cell cycle [19]. In this study, we also found that cells transfected with SARS-COV-2 N protein usually had a higher TUBA1C expression. The SARS-COV-2 N might block host cell G1/S phase through up-regulated TUBA1 expression. By the way, TUBB6, as one of the β-tubulins, was also found to be highly expressed in SARS-COV-2 N protein transfected cells which might participate in host cell cycle regulation [20]. However, much more studies were needed in this area.

The result of SARS-COV-2 N protein aliphatic index indicated its poor heat resistance which might be good news on SARS-COV-2 prevention. Unfortunately, since SARS and MERS epidemic, lots of anti-CoV agents have been developed against virus proteases, polymerases, MTases and entry proteins. None of them have been proved in clinical therapy [21, 22]. The herpes simplex virus type 1 (HSV-1) phosphorylation site is S187. After mutation of this site to alanine, the replication ability and virulence level of HSV-1 in mouse central nervous system decreased significantly [23]. Moreover, the influenza C virus replication was significantly lower than that of wild-type recombinant influenza C virus when its phosphorylation site of the second membrane protein at position 78 and/or 103 was replaced with an alanine residue [24]. The coronavirus N protein shows least variation in the gene sequence, therefore indicating it to be a genetically stable protein, which is a primary requirement for an efficient drug target candidate [25]. Through X-ray crystallography analysis, studies had reported that the N-terminal domain of SARS and MERS structurally adjacent to the receptor binding region which might be a promising target for neutralizing antibodies [26]. In this study, the tertiary structure model with potential phosphorylation sites of SARS-COV-2 N protein was built which would promisingly assist the area for further drug exploration and development of recombinant virus vaccine.

However, for the limitation of our laboratory safety level, there were still some unfortunate limitations in this study. Though the SARS-COV-2 tertiary structure model was successfully built, the Verify 3D and Procheck score of SAVES v5.0 evaluation system were not good enough which a further improvement should be made on its tertiary structure. By the way, though N protein shows least variation in the gene sequence, the main secondary structure of random curl and its instability also made difficulties on future studies.

## Conclusion

On general, we primary analyzed the SARS-COV-2 N protein physicochemical property, subcellular localization, protein structure. A total number of 12 SARS-COV-2 N protein phosphorylated sites and 9 potential protein kinase were also found in this study which showed a promising target for further drug exploration and development of recombinant virus vaccine. More studies are needed in SARS-COV-2 N protein.

## Material and methods

### The sequence and location of SARS-COV-2 N protein

The DNA and protein sequence were downloaded from the NCBI (YP_009724397.2) which encoding the SARS-COV-2 N protein was cloned into pET28a-N plasmid and successfully expressed in *E. coli* by New Testing Technology Center of Guangdong Experimental Animal Monitoring Institute (supplementary Fig. 1). The plasmid will be available free of charge for scientific research on SARS-COV-2 (https://jinshuju.net/f/9BnU6j). The NCBI protein-blast was used to compare the SARS-COV-2 N sequence with SARS-CoV and Middle East respiratory syndrome-related coronavirus (MERS). PSORT II Prediction was utilized to predict protein subcellular localization in human cells.

### Western-blot of SARS-COV-2 N protein

Hct-116 Cells transfected with SARS-COV-2 N plasmid and negative control was harvested and extracted total proteins.. Then, the protein concentration was quantified using a BCA protein assay kit (Beyotime, Shanghai, China). Sodium dodecyl sulfate (SDS)-polyacrylamide gel electrophoresis and Western blot analyses were performed according to the standard procedures. Next, the gel was stained with Coomassie Brilliant Blue for 1 h and decolorized overnight.

### Mass spectrometry analysis

Hct-116 Cells treated with SARS-COV-2 N plasmid and negative control was harvested and extracted total proteins. Then, the protein concentration was quantified using a BCA protein assay kit (Beyotime, Shanghai, China). Western-blot was further used to separate the proteins. Then the N protein complexes were denatured, reduced, alkylated and digested with immobilized trypsin (Promega) for mass spectrometry analysis.

### Cell cycle test

Cell cycle kit (Keygentec KGA511, China) was used in this study. According to the kit instructions, Cells were digested using 0.1% tryps in without EDTA and centrifuged at 1000 rpm. Then binding buffer was used to suspend cells, keeping cell concentration at 1 × 10^6^ cells/mL. Remove the supernatant, add 500ul of cold 70% ethanol to fix the cells (2 h to overnight), store at 4 °C, wash off the fixative with PBS before staining; Add 500 μL PI/RNase A staining working solution and avoid light at room temperature for 30-60 min. After the incubation, cell cycle was detected using flow cytometry within 1 h.

### The physicochemical properties of SARS-COV-2 N protein

The chemical formula, number of amino acids, molecular weight, theoretical pI, number of charged residues, estimated half-life, instability index, aliphatic index using and grand average of hydropathicity (GRAVY) was analyzed by ProtParam tool [27]. A protein with GRAVY > 0 was defined as hydrophobic protein and a protein GRAVY< 0 was defined as hydrophilic protein. Aliphatic index< 70 was defined as poor heat resistance. GRAVY > 0 was defined as hydrophobic protein and GRAVY< 0 was defined as hydrophilic protein.

### The structure of SARS-COV-2 N protein

The SOPMA tool was firstly applied to analyze the secondary structure of SARS-COV-2 N protein [28]. Next we used Swiss-model to generate tertiary structure [29]. For models with less than 100 residues, the sequence identity must be over 30%. For models with greater than 100 residues the QMEAN score must be greater than − 5 [30]. QMEAN Z-scores around zero suggested a good agreement between the model structure and experimental structures of similar size. Scores of − 5.0 or below are an indication of models with low quality. The GMQE (Global Model Quality Estimation) is expressed as a number between 0 and 1, reflecting the expected accuracy of a model built with that alignment and template and the coverage of the target. Higher numbers indicate higher reliability. Finally, SAVES v5.0 which provides quality measures for protein crystal structure and uses five different online tools such as WHATCHECK, PROCHECK, ERRAT, Verify3D, PROVE to assess the quality of the predicted 3D model of SARS-COV-2 N protein [31].

### The SARS-COV-2 N protein phosphorylated sites prediction

The SARS-COV-2 N protein sequence was up-loaded to NetPhos3.1 Server to analyze the potential phosphorylation sites [32]. The prediction score (a value in the range [0.000–1.000]) which above 0.500 indicated positive predictions. The active kinase or the string “unsp” was represented for non-specific prediction. So those phosphorylated sites with specific predicted kinase were then included in the study. Then PROVEAN PROTEIN was applied to further analyzed whether the phosphorylated site variants would affect the structure and function of the N protein [33].

## Supplementary Information

**Additional file 1: Supplementary Fig. 1.** The blast and western-blot results of SARS-COV-2 N protein. a: the sequence blast between SARS-COV-2 and SARS; b: the sequence blast between SARS-COV-2 and MERS; c: western-blot of SARS-COV-2 protein.

**Additional file 2: Supplementary Fig. 2.** The flow chart and the main tools used in the study.

**Additional file 3: Supplementary Table 1.** The secondary structure comparison of SARS-COV-2, SARS and MERS N protein.

**Additional file 4: Supplementary file 1**. The sequence data of Mass Spectrometry Analysis.

## Data Availability

All the sequence data was provided in Supplementary file 1.
