# Supplementary figures and images for: Identification and functional analysis of the SARS-COV-2 nucleocapsid protein
Source: BMC Microbiol. 2021 Feb 22;21:58. doi: 10.1186/s12866-021-02107-3 (PMC7898026; doi:10.1186/s12866-021-02107-3)

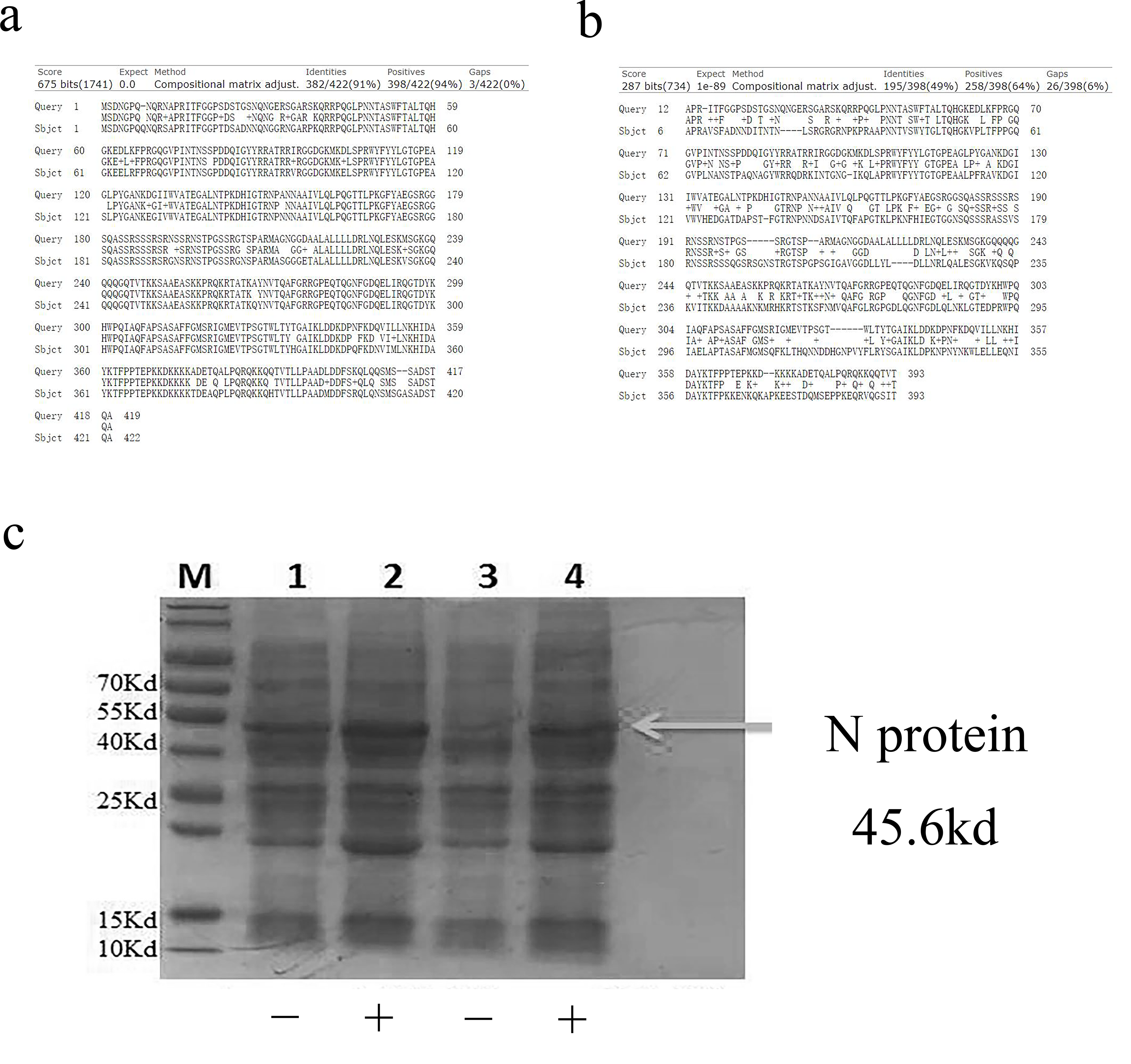

Supplement: Supplementary file 1 — Additional file 1: Supplementary Fig. 1. The blast and western-blot results of SARS-COV-2 N protein. a: the sequence blast between SARS-COV-2 and SARS; b: the sequence blast between SARS-COV-2 and MERS; c: western-blot of SARS-COV-2 protein. [file 12866_2021_2107_MOESM1_ESM.tif]

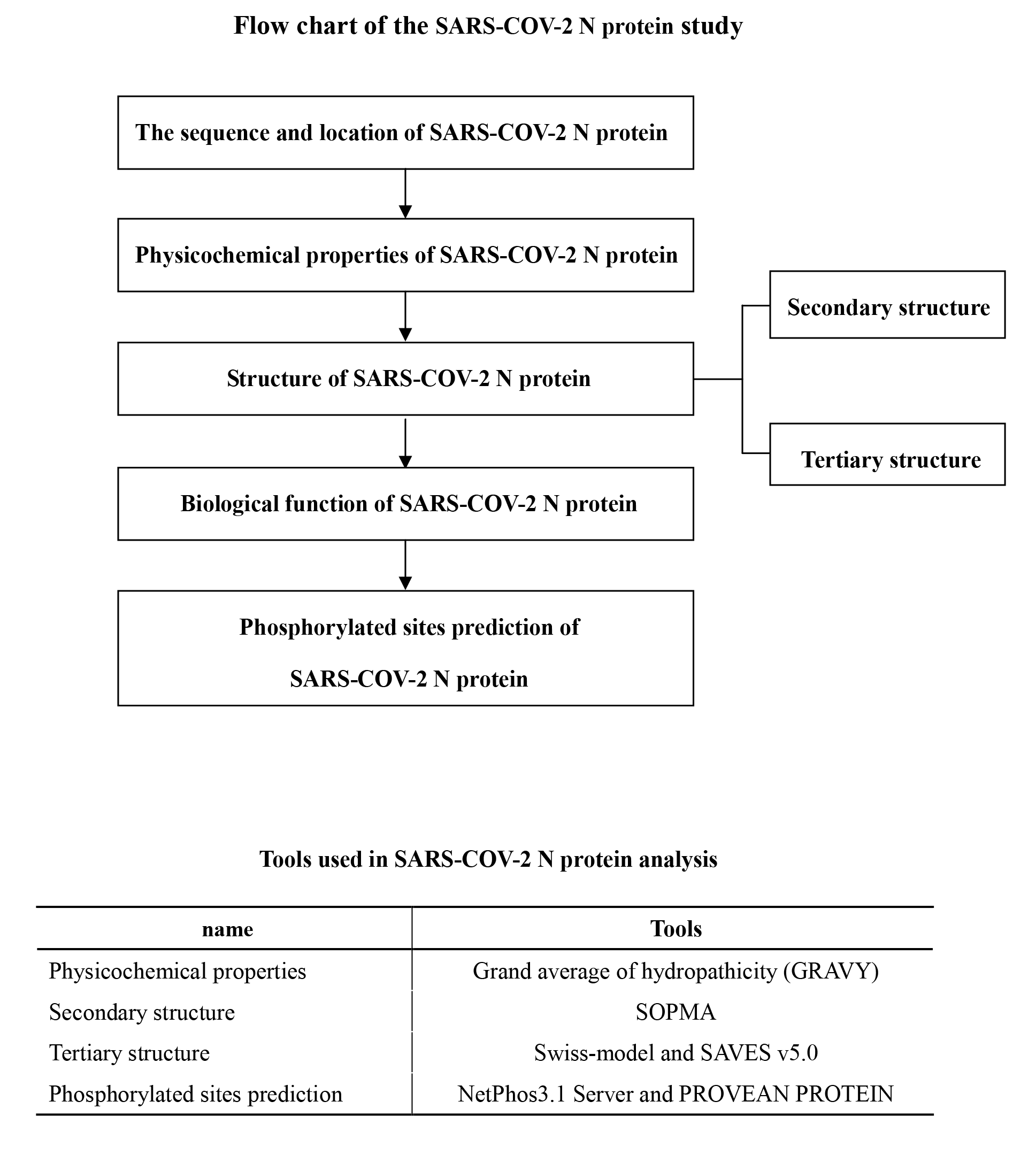

Supplement: Supplementary file 2 — Additional file 2: Supplementary Fig. 2. The flow chart and the main tools used in the study. [file 12866_2021_2107_MOESM2_ESM.tif]
