# Supplementary material for: Identification and functional analysis of the SARS-COV-2 nucleocapsid protein
Source: BMC Microbiol. 2021 Feb 22;21:58. doi: 10.1186/s12866-021-02107-3 (PMC7898026; doi:10.1186/s12866-021-02107-3)
Supplement: Supplementary file 3 — Additional file 3: Supplementary Table 1. The secondary structure comparison of SARS-COV-2, SARS and MERS N protein. [file 12866_2021_2107_MOESM3_ESM.doc]

**Supplementary table 1 the secondary structure comparison of SARS-COV-2, SARS and MERS N protein**

| **Project Name** | **SARS-COV-2** | **SARS** | **MERS** |
| --- | --- | --- | --- |
| **secondary structure** |  |  |  |
| Alpha helix | 89/419 (21.24%) | 86/419(20.53%) | 55/388(14.18%) |
| Extended strand | 70/419 (16.71%) | 75/419(17.90%) | 70/388(18.04%) |
| Beta turn | 29/419 (6.92%) | 25/419(5.97%) | 26/388(6.70%) |
| Random coil | 231/419 (55.13%) | 233/419(55.61%) | 237/388(61.08%) |
